# Biomarker combinations from different modalities predict early disability accumulation in multiple sclerosis

**DOI:** 10.3389/fimmu.2025.1532660

**Published:** 2025-01-31

**Authors:** Vinzenz Fleischer, Tobias Brummer, Muthuraman Muthuraman, Falk Steffen, Milena Heldt, Maria Protopapa, Muriel Schraad, Gabriel Gonzalez-Escamilla, Sergiu Groppa, Stefan Bittner, Frauke Zipp

**Affiliations:** ^1^ Department of Neurology, Focus Program Translational Neuroscience (FTN), Rhine Main Neuroscience Network (rmn^2^), University Medical Center of the Johannes Gutenberg University Mainz, Mainz, Germany; ^2^ Department of Neurology, Section of Neural Engineering with Signal Analytics and Artificial Intelligence, University Hospital Würzburg, Würzburg, Germany

**Keywords:** multiple sclerosis, biomarker, magnetic resonance imaging, neurofilament, optical coherence tomography, disease progression, prediction, structural equation modeling

## Abstract

**Objective:**

Establishing biomarkers to predict multiple sclerosis (MS) disability accrual has been challenging using a single biomarker approach, likely due to the complex interplay of neuroinflammation and neurodegeneration. Here, we aimed to investigate the prognostic value of single and multimodal biomarker combinations to predict four-year disability progression in patients with MS.

**Methods:**

In total, 111 MS patients were followed up for four years to track disability accumulation based on the Expanded Disability Status Scale (EDSS). Three clinically relevant modalities (MRI, OCT and blood serum) served as sources of potential predictors for disease worsening. Two key measures from each modality were determined and related to subsequent disability progression: lesion volume (LV), gray matter volume (GMV), retinal nerve fiber layer, ganglion cell-inner plexiform layer, serum neurofilament light chain (sNfL) and serum glial fibrillary acidic protein. First, receiver operator characteristic (ROC) analyses were performed to identify the discriminative power of individual biomarkers and their combinations. Second, we applied structural equation modeling (SEM) to the single biomarkers in order to determine their causal inter-relationships.

**Results:**

Baseline GMV on its own allowed identification of subsequent EDSS progression based on ROC analysis. All other individual baseline biomarkers were unable to discriminate between progressive and non-progressive patients on their own. When comparing all possible biomarker combinations, the tripartite combination of MRI, OCT and blood biomarkers achieved the highest discriminative accuracy. Finally, predictive causal modeling identified that LV mediates significant parts of the effect of GMV and sNfL on disability progression.

**Conclusion:**

Multimodal biomarkers, i.e. different major surrogates for pathology derived from MRI, OCT and blood, inform about different parts of the disease pathology leading to clinical progression.

## Introduction

In multiple sclerosis (MS), disability progression is closely related to neuroaxonal degeneration (1, 2). Therefore, identifying and quantifying axonal damage is an essential step towards improved clinical decision-making and prognostication. Currently, magnetic resonance imaging (MRI) is the most established non-invasive modality for diagnosing, evaluating treatment effectiveness, and monitoring disease progression in patients with MS. In particular, conventional structural MRI metrics, like T2-hyperintense lesion volume (LV) and gray matter volume (GMV), have been proven to be reproducible and well-validated in reflecting disease activity and progression, respectively (3, 4). However, recent technical advances, such as single molecule array (SiMoA) and easily accessible optical coherence tomography (OCT), have enabled additional non-invasive measurements of neurodegeneration-related biomarkers with increasing clinical application (5, 6). Therefore, blood-based biomarkers such as serum neurofilament light chain (sNfL) and serum glial fibrillary acidic protein (sGFAP), as well as measures of retinal thickness (retinal nerve fiber layer (RNFL), ganglion cell inner plexiform layer (GCIPL)) have gained significant interest for diagnostic purposes and are expected to be applied in clinical routine soon.

Nevertheless, all biomarkers have certain limitations due to the nature of their respective modalities: MRI is most effective at detecting focal white matter lesions in the brain and spinal cord, but lesions in gray matter structures can only be reliably visualized with rather high field strengths (7). Additionally, conventional MRI is functionally “blind” to what is known as “normal-appearing white matter” (NAWM). Blood biomarkers of neuronal (sNfL) or glial (sGFAP) damage can be influenced by different factors such as age, blood volume, genetics, and other medical conditions such as impaired renal function (8–10). Additionally, measures of retinal thickness may not always accurately reflect the presence and extent of inflammation or damage in the brain and spinal cord, as they may be affected by factors such as pupil dilation, eye movements, and the presence of cataracts or other eye conditions, which can impact the accuracy of the results (6, 11). Furthermore, the spatial resolution is limited, as OCT captures only a small part of the central nervous system (CNS). Thus, the concept of “one biomarker” indicating the existence of an underlying disease-specific process remains a utopia in predicting disease progression. However, individual challenges may be overcome by combining biomarkers from different modalities that ideally also represent multiple aspects of MS pathology.

Utilizing multiple biomarkers from different modalities has already been demonstrated in other neurological disorders such as Alzheimer’s disease, where a combination of positron emission tomography (PET)-imaging and cerebrospinal fluid (CSF) biomarkers has enabled a more precise diagnostic evaluation (12, 13). In people with MS, initial efforts have shown that multimodal biomarkers can predict neuropsychological parameters such as cognitive impairment (14). However, it is unclear which biomarker combinations offer the best discriminative accuracy for disease progression of MS. The combination of several biomarkers altogether, by means of predictive modeling, may be able to compile large amounts of multimodal data, in order to attain solid conclusions and decision making in MS monitoring.

Thus, the aim of this study was to investigate the prognostic value of individual biomarkers (MRI, OCT and blood), as well as their combinations in predicting four-year disease activity and progression in MS. To test this, we determined LV and GMV from MRI, RNFL and GCIPL from OCT and sGFAP and sNfL from blood within a cohort of 111 MS patients who were clinically followed up for four years.

## Methods

### Participants

**Figure 1:**
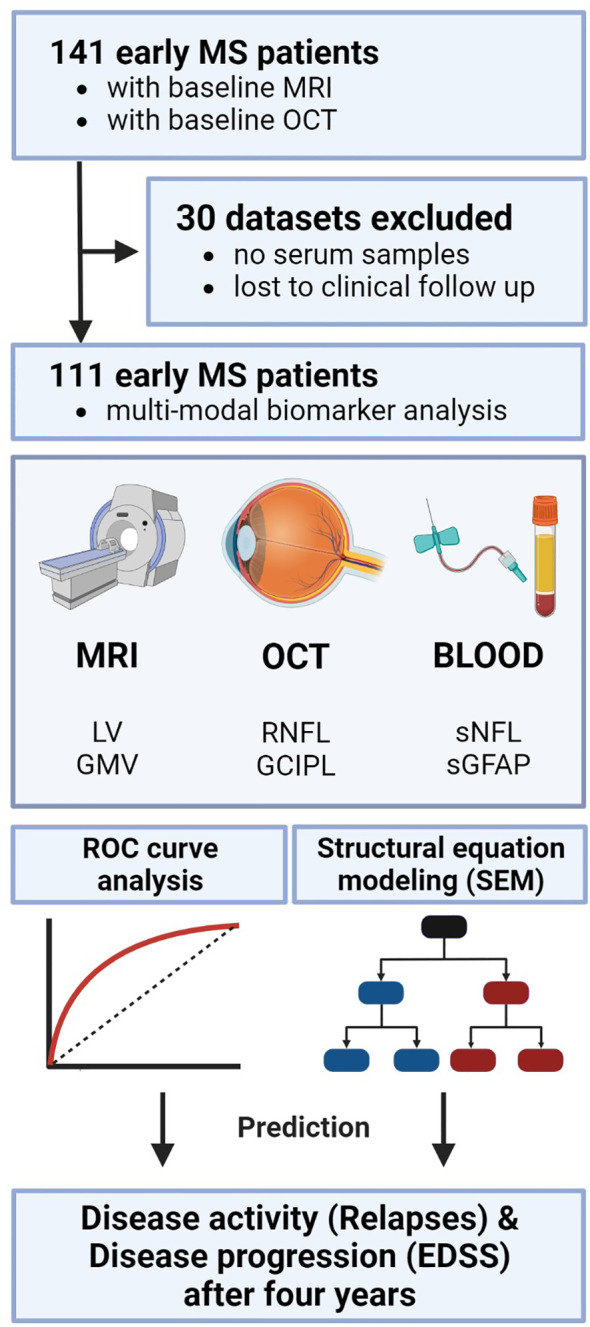
Study analysis design. Study protocol and design including the three modalities each with two biomarkers as potential predictors. Statistically, ROC analysis was performed to evaluate the discriminative power of single and combined biomarkers. Subsequently, SEM was applied to test the causal inter-relationships between the variables. EDSS, expanded disability status scale; GCIPL, ganglion cell-inner plexiform layer; GMV, gray matter volume; OCT, optical coherence tomography; RNFL, retinal nerve fiber layer; ROC, receiver operator characteristics; SEM, structural equation modeling; sGFAP, serum glial fibrillary acidic protein; sNfL, serum neurofilament light; LV, lesion volume.

In total, out of 141 MS patients that were retrospectively screened for this project, 111 MS patients that underwent a comprehensive and detailed clinical assessment were finally included in the analysis (**Figure 1**). The selected cohort included MS patients with MRI (T2-hyperintense LV and GMV), blood (sNfL and sGFAP), and OCT (RNFL and GCIPL) measurements at the outpatient clinic of the Department of Neurology, at the University Medical Center Mainz (Germany) (**Table 1**). All included patients had relapsing-remitting multiple sclerosis (RRMS) as diagnosed according to the 2017 revised McDonald diagnostic criteria (15). The mean (± standard deviation) disease duration of all patients at study inclusion was 3.15 ± 4.26 years. All diagnostic baseline measurements were performed within 6 months of study inclusion. An experienced neurologist clinically assessed patients and their Expanded Disability Status Scale (EDSS) score at study entry and follow up visit (3.74 ± 1.25 years), along with clinical relapse history over the study period. EDSS progression was defined as an increase of ≥ 1 point in the EDSS score for a baseline score of ≥ 1.5 or a 1.5 point increase for a baseline score of 0 (16). A clinical relapse was defined as a monophasic clinical episode with new neurological symptoms, lasting more than 24 h and in the absence of fever or infection (15). The annualized relapse rates (ARR) were calculated by dividing the total number of all observed relapses by the total number of patient-years. All measurements were performed at least 30 days after a clinical relapse and/or a high-dose corticosteroid treatment.

**Table 1:** Basic characteristics. Demographic and clinical data of the included MS patients as well as MRI, OCT and blood biomarkers at baseline.

Demographics	MS patients (n = 111)
Age [years] mean ± SD	34.8 ± 9.67
Sex [female] (percent)	79 (71)
Disease duration [years] mean ± SD	3.15 ± 4.26
Disease-modifying treatment	
None (percent)	18 (16)
Mild to moderate efficacy (percent)	69 (62)
High efficacy (percent)	24 (22)
Clinical measures	
Baseline EDSS median (25^th^; 75^th^ percentile)	1.0 (0.0; 2.0)
Follow up EDSS median (25^th^; 75^th^ percentile)	1.5 (0.0; 2.5)
Patients with EDSS progression (percent)	46 (41.4)
Relapses over 4 years mean ± SD	0.76 ± 1.17
Annualized relapse rate mean ± SD	0.21 ± 0.33
Time to follow up [years] mean ± SD	3.74 ± 1.25
Patients with history of optic neuritis (percent)	33 (30)
MRI measures	
LV [ml] mean ± SD	5.97 ± 9.57
GMV [fraction] mean ± SD	0.43 ± 0.03
OCT measures	
RNFL [mm^3^] mean ± SD	0.21 ± 0.02
GCIPL [mm^3^] mean ± SD	0.76 ± 0.1
Blood measures	
sNfL [z-score] mean ± SD	0.115 ± 2.21
sGFAP [pg/ml] mean ± SD	121.2 ± 43.8

Mild to moderate efficacy = interferons, glatiramer acetate, teriflunomide, dimethyl fumarate.

High efficacy = natalizumab, anti-CD20 monoclonal antibodies, sphingosine-1-phosphate receptor modulators, alemtuzumab.

EDSS, extended disability status scale; GCIPL, ganglion cell-inner plexiform layer; GMV, gray matter volume; LV, lesion volume; MRI, magnetic resonance imaging; OCT, optical coherence tomography; RNFL, retinal nerve fiber layer; SD, standard deviation; sGFAP, serum glial fibrillary acidic protein; sNfL, serum neurofilament light.

### sNfL and sGFAP measurements

Serum samples were collected by attending physicians at the University Medical Center Mainz. Samples were processed at room temperature within 2 hours. Serum samples were spun at 2000xg at room temperature for 10 minutes, aliquoted in polypropylene tubes and stored at −80°C. sNfL and sGFAP concentrations were measured as previously described (10, 14). In brief, sNfL and sGFAP levels were determined using the highly sensitive single molecule array (SiMoA) technology (17). Samples were measured in duplicates by SiMoA HD-1 (Quanterix, USA) using NF-Light Advantage kits according to the manufacturer’s instructions. The mean inter-assay and intra-assay coefficient of variation was less than 10%. Measurements were performed in a blinded fashion without information about clinical data.

### MRI data acquisition

MRI data acquisition was performed as previously described (14). In brief, structural MRI was performed on a 3-Tesla MRI scanner (Magnetom Tim Trio, Siemens, Germany) with a 32-channel receive-only head coil. In all patients, imaging was performed using a sagittal 3D T1-weighted magnetization-prepared rapid gradient echo (MP-RAGE) sequence (TE/TI/TR = 2.52/900/1900 ms, flip angle = 9°, field of view = 256 × 256 mm2, matrix size = 256 × 256, slab thickness = 192 mm, voxel size = 1 × 1 × 1 mm^3^) and a sagittal 3D T2-weighted fluid-attenuated inversion recovery (FLAIR) sequence (TE/TI/TR = 388/1800/5000 ms, echo-train length = 848, field of view = 256 × 256 mm^2^, matrix size = 256 × 256, slab thickness = 192 mm, voxel size = 1 × 1 × 1 mm^3^). A clinician scientist blinded to the patient data excluded major anatomical abnormalities based on the subject’s T1-weighted and FLAIR images of the whole brain.

### Quantification of white matter LV and GMV

The quantification of WM (white matter) volume, lesion volume and GMV was performed as previously described (14). Using voxel-based morphometry (VBM) analysis in the Statistical Parametric Mapping (SPM8) software, the GM and WM volumes were calculated. The volumes of WM lesions were assessed using the cross-sectional lesion growth algorithm of the lesion segmentation toolbox (18) included in the SPM8 software. 3D FLAIR images were co-registered to 3D T1-weighted images and bias corrected. After partial volume estimation, lesion segmentation was performed with 20 different initial threshold values for the lesion growth algorithm (18). By comparing manually and automatically estimated lesion maps, the optimal threshold (ĸ value, dependent on image contrast) was determined, and average values were calculated for each patient. A uniform ĸ value of 0.1 was applied in all patients in order to automatically estimate lesion volume and filling of 3D T1-weighted images. Subsequently, the filled 3D T1-weighted images and the native 3D T1-weighted images were segmented into GM, WM, and CSF and then normalized to the Montreal Neurological Institute (MNI) space. The quality of the segmentations was visually inspected to increase reliability.

### OCT: image acquisition and scanning protocol

The analysis was performed as previously described (19, 20). In brief, the Advised Protocol for OCT Study Terminology and Elements (APOSTEL) recommendations were followed (21) including a quality control for the raw OCT scans complying with the OSCAR-IB criteria (22). MS patients with accompanying diseases potentially affecting the optic nerve or other ocular disease were excluded in advance. Hence, none of the patients had a history of glaucoma, retinopathy or other neurological disorders (besides RRMS). An experienced operator performed OCT image acquisition following a unified standard acquisition protocol using a spectral domain OCT (Heidelberg Spectralis, Heidelberg Engineering, Germany) with Heidelberg Eye Explorer software (HEYEX, version 1.10.2.0). The measurements were acquired in a shaded room at ambient light without pupillary dilation. Intra-retinal layers of the macula were gauged by a standardized scan comprising 61 vertical or horizontal B-scans while focusing on the fovea at a scanning angle of 30° × 25° and a resolution of 768 × 496 pixels. Automatic real time was set to nine at high-speed scanning mode. Confocal scanning laser ophthalmoscopy was performed in parallel and revealed no evidence of pathology. No further fundoscopic imaging was carried out. To account for inter-eye within-patient dependencies, we calculated the mean of both eyes in patients with no history of optic neuritis; in patients with a history of unilateral optic neuritis, we only used the OCT scan of the non-affected eye. Hence, the main statistical analysis was performed at a per-patient level. All B-scans were automatically segmented (followed by manual correction by a trained rater) using segmentation beta-software (Spectralis Viewing Module version 6.9.5.0) of the Heidelberg Eye Explorer (version 1.10.2.0) provided by the manufacturer. The segmentation lines were the following retinal layers: RNFL, GCIPL, inner nuclear layer, outer plexiform layer and outer nuclear layer. The mean volume of the individual retinal layers was computed in an area of a radius of 3.45 mm around the fovea including the fovea using the Early Treatment of Diabetic Retinopathy Study (ETDRS) grid. Lastly, RNFL and GCIPL were finally selected as primary estimate for neuroaxonal damage of the retina, as both have been associated with brain atrophy and disability worsening (23, 24).

### Statistics

Statistical analysis was performed using SPSS 23 (SPSS, Chicago, IL, USA), MedCalc (Version 20.115) and GraphPad Prism 9 software. Summary statistics are presented as mean ± standard deviation (SD), or median (25^th^ and 75^th^ percentile), or number (percentage), where applicable. To create a combined variable for each biomarker combination, a binary logistic regression model for each combination (corrected for sex, age, disease duration and disease-modifying treatment) was estimated in order to get the predicted probability from each model. Then, we used this probability as the test variable in the subsequent receiver operating characteristic (ROC) procedure (14).

A ROC analysis was performed to calculate the predictive discriminating values for each biomarker and the combinations. This statistical method is preferentially used to make a series of discriminations into two different states based on a specific diagnostic variable. Here, the presence or absence of relapses or EDSS worsening, served as binary classifiers. Every value of that discriminating variable is used as a cut-off with calculation of the corresponding sensitivity and specificity.

### Structural equation modeling

The analysis was performed as previously described (25) using the SEM toolbox for MATLAB (version 13a; Mathworks, Natick, MA, USA). SEM represents a statistical technique that is used to test and estimate structural relationships between variables in a model. By structural, we mean that we incorporate causal assumptions as part of the model. Hence, SEM represents a multivariate technique that is able test complex relationships among multiple variables simultaneously, and estimate the strength and direction of these relationships. In our model, we explored the association between multimodal biomarkers and the clinical outcomes (clinical relapses and EDSS progression). We used the Maximum likelihood method of estimation to fit the models. In order to adjust the models for a large sample size, we used the Root Mean Square Error of Approximation (RMSEA) index, which improves precision without increasing bias (26). The RMSEA index estimates lack of fit in a model compared to a perfect model and therefore should be low. In all models, the Invariant under a Constant Scaling (ICS) and ICS factor (ICSF) criteria should be close to zero, indicating that models were appropriate for analysis. Finally, based on the Akaike Information Criterion (AIC) the quality of each model relative to other models was estimated, with smaller values signifying a better fit of the model. The strength of associations between the variables in the models was quantified by standardized coefficients (s), ranging from 0 (no association) to 1 (very strong association). To correct for potential confounders the models were adjusted for sex, age, disease duration and disease-modifying treatment (DMT). P-values less than 0.05 were considered statistically significant.

## Results

### Patient characteristics

All demographics and clinical characteristics of the investigated cohort are summarized in **Table 1**. In total, 141 early MS patients with baseline MRI and OCT were selected. Thirty patients were excluded from the final analysis because either there was no serum sample available or they were lost to clinical follow-up (**Figure 1**). The mean follow-up time in our longitudinal cohort of 111 patients was 3.74 ± 1.25 years. The mean age ± SD was 34.8 ± 9.67 years; 79 patients (71.0%) were female and 32 (29.0%) were male. The mean disease duration at study inclusion was 3.15 ± 4.26 years. All patients had a relapsing-remitting disease course (RRMS) according to the 2017 revised McDonald criteria (15). At the time of inclusion, 18 patients (16%) were not receiving any DMT, 69 (62%) were receiving a mild to moderate efficacy DMT, and 24 (22%) were receiving a high efficacy DMT. The median baseline disability, quantified with EDSS, was 1.0 (25th and 75th percentile: 0.0−2.0). Overall, 46 patients (41.4%) experienced EDSS progression during the observation period. The mean ARR was 0.21 ± 0.33; 33 (30%) patients had a history of optic neuritis. The results from blood biomarker, MRI, and OCT measurements are also summarized in **Table 1**.

### Predictive discrimination model

**Figure 2:**
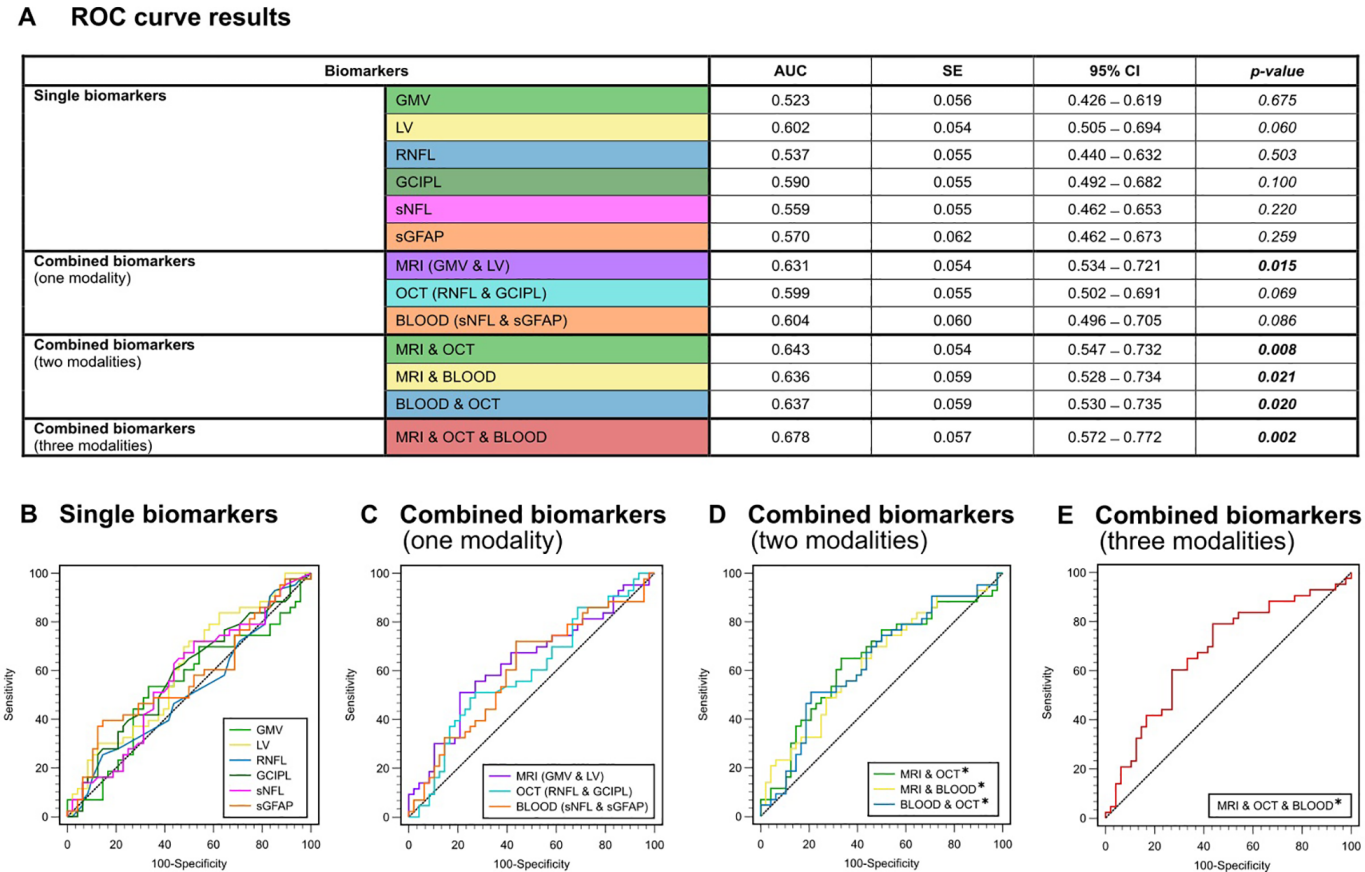
ROC analysis for the discrimination between the presence or absence of relapse activity **(A)** Color-coded table depicting the ROC analysis for individual and combinations of biomarkers. AUC, p-value and 95%-CI for the prediction of clinical relapses (yes/no). **(B)** ROC curves for single biomarkers. **(C)** ROC curves for combined biomarkers within one modality. **(D)** ROC curves for combined biomarkers within two modalities. **(E)** ROC curve for combined biomarkers of all three modalities (GMV + LV, RNFL + GCIPL and sNfL + sGFAP). AUC, area under the curve; CI, confidence interval; GCIPL, ganglion cell-inner plexiform layer; GMV, gray matter volume; LV, lesion volume; OCT, optical coherence tomography; RNFL, retinal nerve fiber layer; ROC, receiver operator characteristics; SE, standard error; sGFAP, serum glial fibrillary acidic protein; sNfL, serum neurofilament light.

**Figure 3:**
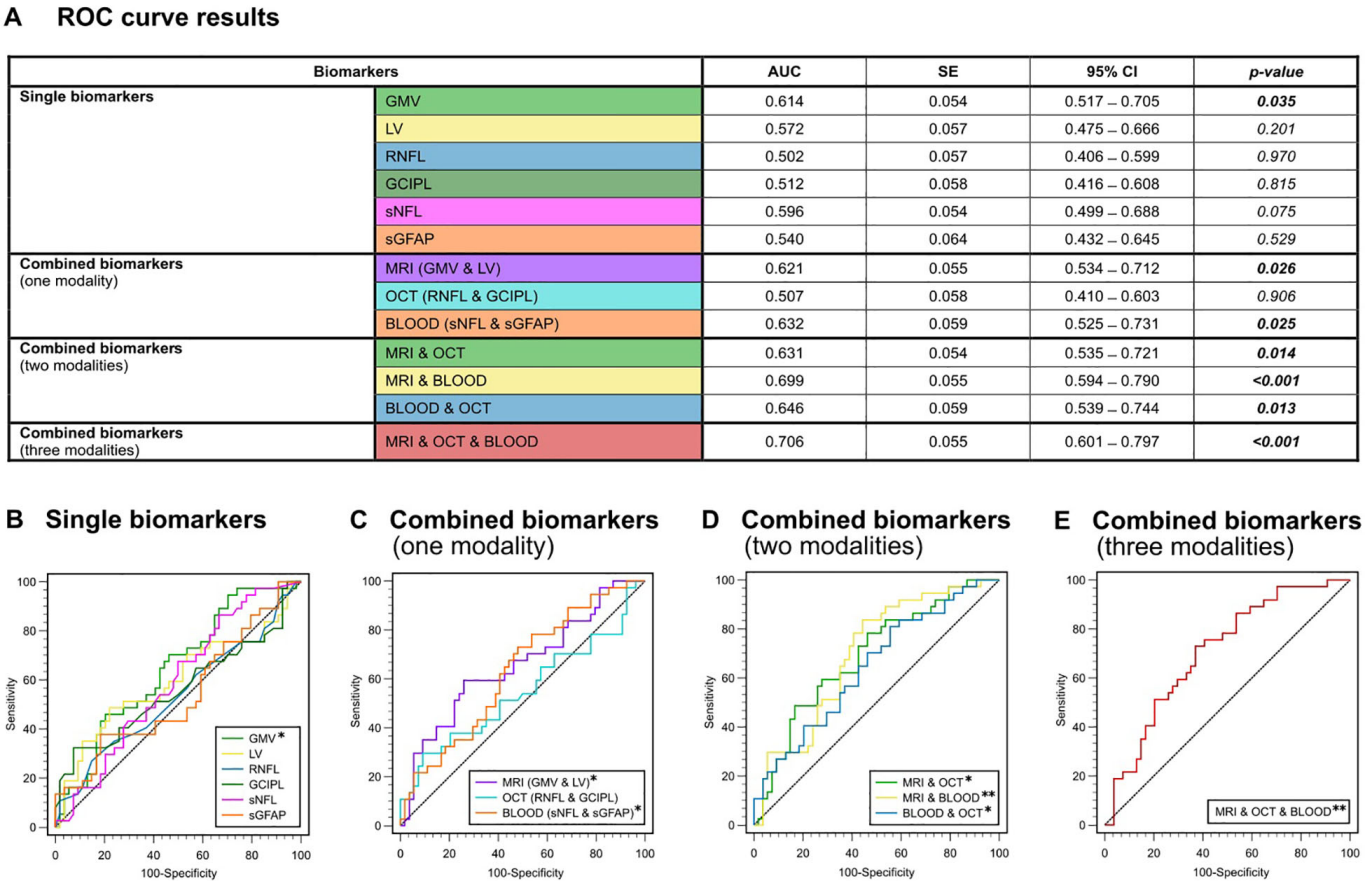
ROC analysis for the discrimination between the presence or absence of EDSS progression. **(A)** Color-coded table depicting the ROC analysis for individual and combinations of biomarkers. AUC, p-value and 95%-CI for the prediction of EDSS progression (yes/no). **(B)** ROC curves for single biomarkers. **(C)** ROC curves for combined biomarkers within one modality. **(D)** ROC curves for combined biomarkers within two modalities. **(E)** ROC curve for combined biomarkers of all three modalities (GMV + LV, RNFL + GCIPL and sNfL + sGFAP). AUC, area under the curve; CI, confidence interval; GCIPL, ganglion cell-inner plexiform layer; GMV, gray matter volume; OCT, optical coherence tomography; RNFL, retinal nerve fiber layer; ROC, receiver operator characteristics; SE, standard error; sGFAP, serum glial fibrillary acidic protein; sNfL, serum neurofilament light; LV, lesion volume.

An overall ROC analysis was performed to determine the predictive discriminating value of the individual and combined measures to distinguish MS patients with and without disease activity (determined through the presence or absence of relapses during this time) and with and without disability progression (determined through the presence or absence of EDSS worsening over four years). Resulting values with AUC, standard error, 95% confidence interval and p-values are presented in detail in **Figures 2A** and **3A**.

In general, none of the individual biomarkers were able to predict the occurrence of clinical relapses within the 4-year observation period (AUC-range: 0.523 – 0.602). All p-values for testing AUC = 0.5 vs. AUC ≠ 0.5 were greater than 0.05 and were hence not significantly different from a random classifier (**Figure 2B**). Only LV showed a trend towards significance (AUC = 0.602; p = 0.060). In the ROC analysis based on the presence or absence of EDSS progression, GMV was the only single biomarker to show significant predictive capability for EDSS progression on its own (AUC = 0.614, SE = 0.054; p = 0.035), whereas all other single biomarkers did not (AUC-range = 0.502 - 0.596) (**Figure 3B**).

When we combined biomarkers within their respective modality, MRI markers (LV + GMV) were able to predict both relapses (AUC = 0.631, SE = 0.054; p = 0.015) and EDSS progression over the four-year period (AUC = 0.621, SE = 0.055; p = 0.026). Combined blood biomarkers (sNfL + sGFAP) were only able to predict EDSS progression (AUC = 0.632, SE = 0.059; p = 0.025), while combined OCT measures (RNFL + GCIPL) were unable to predict either clinical relapses (AUC = 0.599, SE = 0.054; p = 0.069) or EDSS progression (AUC = 0.507, SE = 0.058, p = 0.906) (**Figures 2C**, **3C**).

However, all combinations of two biomarker modalities significantly predicted clinical relapses (AUC range = 0.636 – 0.643) and EDSS progression (AUC range = 0.631 – 0.699) (**Figures 2D**, **3D**). The best prediction for EDSS progression using two modalities was achieved with a combination of MRI and blood biomarkers (AUC = 0.699, SE = 0.055; p < 0.001).

Most notably, the combination of all six biomarkers achieved the highest AUC for discriminating MS patients with clinical relapse activity from those without (AUC = 0.678, SE = 0.057; p = 0.002) and for discriminating progressive from non-progressive MS patients (AUC = 0.706, SE = 0.055; p < 0.001) (**Figures 2E**, **3E**). Overall, these results demonstrate that the predictive capability of single biomarkers remains limited except for GMV, whereas combining multimodal biomarkers stepwise improves their accuracy in prediction of both relapse activity and disease progression within early multiple sclerosis.

### MRI and blood biomarkers influence disease activity and progression

**Figure 4:**
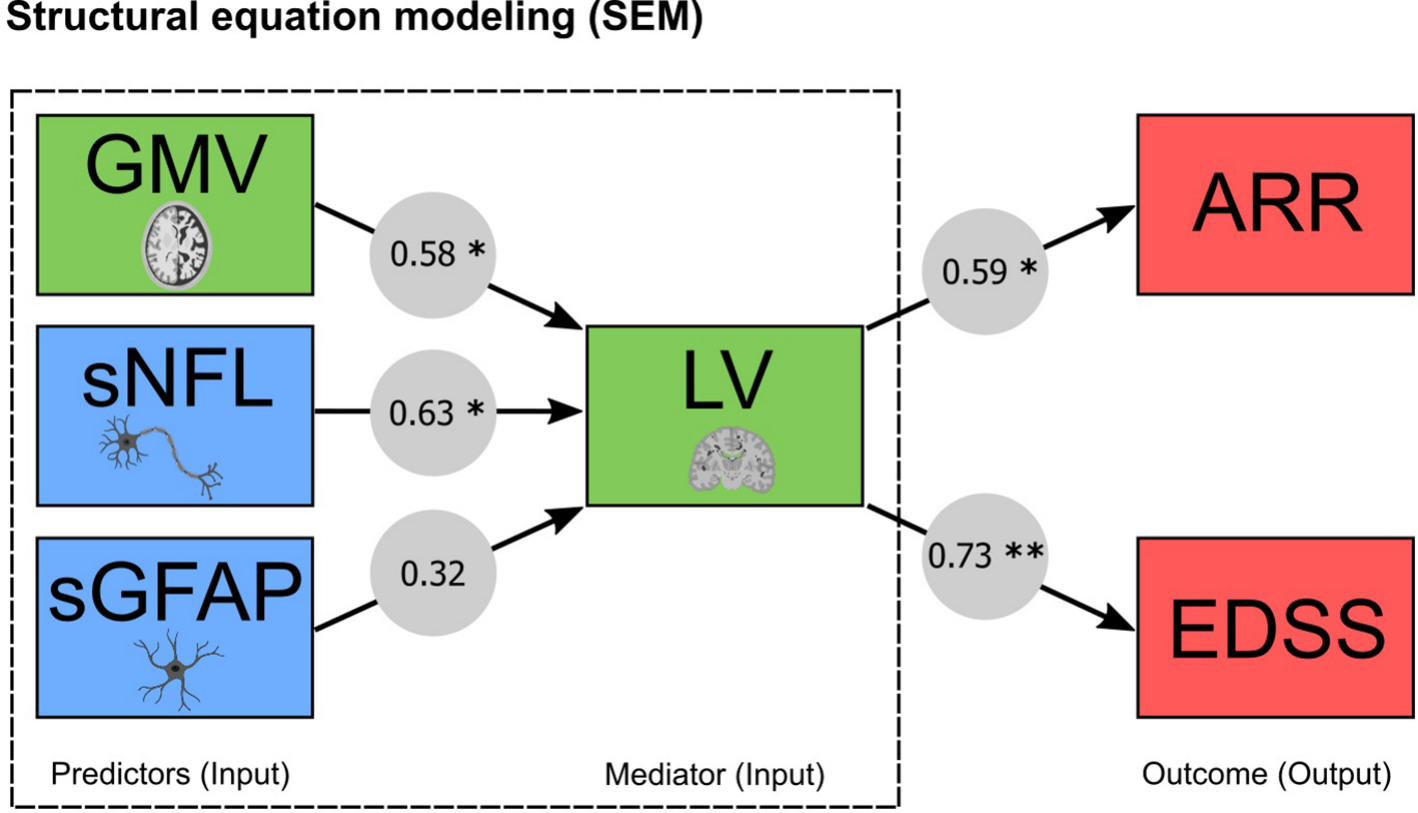
MRI and blood biomarkers and their capability to predict clinical outcomes through structural equation modeling (SEM). Predictive modeling of MRI (GMV and LV) and blood (sNfL and sGFAP) biomarkers. Arrows denote the relationship between the variables expressed as standardized coefficients, which are shown for each path (* significant at p < 0.01; ** significant at p < 0.001). ARR, annualized relapse rate; EDSS, expanded disability status scale; GMV, gray matter volume; sGFAP, serum glial fibrillary acidic protein; sNfL, serum neurofilament light; LV, lesion volume.

In order to create a prediction model analyzing complex relationships among multiple variables, we next applied SEM to assess the causal relationship of the most promising biomarker combinations determined in the ROC approach, namely MRI (LV + GMV) and blood (sNfL + sGFAP) biomarkers. In addition to the ROC analysis, SEM allows us to test a model for its compatibility with the data in its entirety simultaneously. In the predictive modeling approach, the RMSEA index for the models was below 0.03 and the AIC comparing the models varied between 0.006 and 0.019. The obtained fit indices in the SEM analysis implied a good fit of the constructed models to the observed data, providing robust relations between the variables. Within the SEM model quantifying the pathways, the input variables (GMV, sNfL, sGFAP and LV) predicted both ARR and EDSS progression. Our model with resultant standardized coefficients (s) identified that GMV (s = 0.58; p < 0.01) and sNfL (s = 0.63; p < 0.01) significantly predict ARR and EDSS progression through lesion volume as mediator (ARR [s = 0.59; p < 0.01] and EDSS [s = 0.73; p < 0.001]) (**Figure 4**). Taken together, LV mediates the path between GMV and sNfL on the one side, and ARR and EDSS progression on the other side.

## Discussion

Here, we present a longitudinal study utilizing a classification model and a multivariate analysis technique to predict both disease activity and progression in patients with early MS based on multimodal biomarker combinations. In our discrimination model, the triple combination of MRI (LV and GMV), OCT (RNFL and GCIPL) and blood biomarkers (sNfL and sGFAP) achieved the best performance in predicting disability progression as well as disease activity within the upcoming four years. Our subsequently constructed SEM model established sNfL, GMV and LV as viable predictors of both disease activity and progression. Beyond that, the model further indicated that LV significantly mediates the effect of sNfL and GMV on future disease activity and progression over the study period. Thereby, our multi-biomarker approach highlights the importance of accounting for LV (neuroinflammation) when implementing cross-modal biomarkers in predicting clinical outcomes in MS.

Our findings align well with the current understanding of the pathophysiology in early, inflammation-driven MS, where disease activity (T2-hyperintense LV) drives ongoing neuroaxonal degeneration (sNfL and GMV) and clinical disability progression (27). Although each biomarker has been found to predict certain aspects of MS pathology individually (6, 17, 28–30), they all have their own individual strengths and weaknesses. In line with this, the predictive ability of each biomarker in our ROC analyses was limited when used on its own, but gained an incremental value when applied in combination with other biomarkers. Importantly, combining biomarkers from different modalities, such as MRI and blood biomarkers, resulted in a significant improvement in predicting both relapse activity and disease progression. This implies that certain biomarkers might be able to compensate for the limitations of others. For example, blood biomarkers have been found to be poor predictors of fatigue in MS (14, 31), while imaging of deep gray matter and brainstem structures have shown strong associations with measures of fatigue (25). Additionally, blood biomarkers provide a holistic view of cellular damage across the entire neuroaxis with high temporal resolution but lack of spatial resolution (5, 8), while conventional MRI markers provide great spatial resolution but are naturally “blind” for slightly injured tissue such as NAWM. Therefore, using both imaging and blood biomarkers can provide a more comprehensive understanding of disease progression in MS, as they can offer complementary information of different aspects of the disease process. Furthermore, the integration of potentially latent variables via observed variables in the characterization of cross-modal biomarkers may help to identify patients at risk of disease progression, and therefore aid therapeutic decision-making. Appropriate biomarkers may even been chosen according to a patient’s individual symptoms and signs, which could allow for the creation of more personalized treatment plans. Accordingly, a recent study found predictors with mid- to high-accuracy for several disability outcomes in MS by combining clinical and imaging with omics information (32). This machine learning study particularly identified algorithms for predicting the escalation of therapy from first-line to high-efficacy treatment.

A plethora of different blood biomarker candidates has been evaluated in clinical and pre-clinical studies on neuroinflammation (33). However, sNfL and more recently sGFAP have shown the greatest prognostic potential in MS (14, 33), therefore, we preselected those biomarkers for our study. There are several surrogate markers of neurodegeneration in MR imaging, such as brain parenchyma fraction, total brain volume, and GMV (34). We decided to primarily include GMV in our analyses since it is widely used and has a strong association with neurodegeneration and cognitive impairment (29, 34). However, as models and algorithms become more complex and advanced, it makes sense to include more biomarkers in order to further improve predictive accuracies. In MS, OCT has been used to detect thinning of retinal layers; this loss of retinal nerve fibers may be indicative of underlying neurodegeneration (6). However, in our early MS cohort, inclusion of OCT did not show a remarkable additive effect in predicting disease progression or relapse rates. This may have several reasons: first, changes in the eyes of our early MS cohort may be subtle and not always be detectable with OCT. Furthermore, although OCT has a good resolution for damage to the visual system, namely the retina and the layers immediately beneath it, as well as the optical radiation, it may not provide sufficient information on neurodegeneration in other regions of the CNS, such as infratentorial structures (6, 11). Additionally, previous studies have shown RNFL to be a significantly variable measure, especially when considering non-optic neuritis eyes (35–37). In line with this, in our cohort, only 33 patients had a history of prior optic neuritis and in order to look at neurodegeneration in MS in general, we only included OCT results from eyes without prior optic neuritis in our analyses. This may have limited the predictive capability of our OCT results; however, both GCIPL and RNFL are well-established markers and have been associated with disease progression even when applied for non-optic neuritis eyes (38).

Our study also has some limitations: First, we investigated a real-world cohort. Hence, the time point for measuring all biomarkers showed some ranges. However, a real-world cohort has the advantage of resembling a more realistic clinical situation and may therefore suffer less from a selection bias (39). Second, longer follow-up observations are warranted. Third, total GM atrophy is related to disability in MS (29, 40), but also regional GM atrophy e.g. thalamic volume plays a key role for clinical progression (41). Finally, also changes within the NAWM are relevant for disease worsening in MS (42, 43). Hence, further studies are needed to incorporate more specific and advanced MRI-derived markers into such multimodal approaches.

Altogether, the combination of multimodal biomarkers (LV, GMV, RNFL, GCIPL, sNfL, sGFAP) that represent different parts of the disease pathology offer advantages in predicting upcoming disability accumulation in MS. In addition, predictive modeling specifically revealed that total lesion volume is a substantial mediator of the prognostic properties of gray matter and neurofilament on future progression indicating the significance of overall cerebral lesion load in fostering neuronal loss and subsequent disability. Validation and replication of multimodal biomarkers identified so far will be required for generating the evidence to be applied in personalized health care for people with MS.

## Data Availability

The datasets presented in this article are not readily available because restrictions apply to the availability of these data, which were used under license for the current study and are therefore not publicly available. The raw data used in preparation of the figures and tables will be shared in anonymized format upon reasonable request by a qualified investigator for purposes of replicating procedures and results. Requests to access the datasets should be directed to vinzenz.fleischer@unimedizin-mainz.de.
